# Implant survival of 2,723 vitamin E-infused highly crosslinked polyethylene liners in total hip arthroplasty: data from the Finnish Arthroplasty Register

**DOI:** 10.1080/17453674.2021.1879513

**Published:** 2021-02-01

**Authors:** Matias Hemmilä, Inari Laaksonen, Markus Matilainen, Antti Eskelinen, Jaason Haapakoski, Ari-Pekka Puhto, Jukka Kettunen, Konsta Pamilo, Keijo T Mäkelä

**Affiliations:** a Department of Orthopaedic Surgery, University of Turku and Turku University Hospital, Turku;; b Turku PET Centre, University of Turku and Turku University Hospital, Turku;; c Coxa Hospital for Joint Replacement and Faculty of Medicine and Health Technologies, Tampere University, Tampere;; d National Institute for Health and Welfare, Helsinki;; e Division of Operative Care, Department of Orthopaedic and Trauma Surgery, Oulu University Hospital, Oulu;; f Department of Orthopaedics and Traumatology, Kuopio University Hospital, Kuopio, Finland

## Abstract

Background and purpose — The use of crosslinked polyethylene in total hip arthroplasty (THA) has decreased wear remarkably. It has been suggested that the antioxidative effects of vitamin E may enhance the wear properties of polyethylene even further. This study evaluates revision rates between vitamin E-infused polyethylene liners (E1 and E-poly, ZimmerBiomet, Warsaw, IN, USA) versus moderately crosslinked polyethylene (ModXLPE) liners from the same manufacturer used in primary THA.

Patients and methods — We conducted a study based on data from the Finnish Arthroplasty Register. The study group consisted of 2,723 THAs with a vitamin E-infused liner and a reference group of 2,707 THAs with a moderately crosslinked polyethylene liner. Survivorship, revision risk, and re-revision causes were compared between groups.

Results — The 7-year survival of the vitamin E-infused polyethylene liner group and of the reference group with revision for any reason as the endpoint was comparable (94% [95% CI 92.9–94.9] and 93% [CI 91.9–93.9], respectively). The adjusted hazard ratio (HR) for any revision was similar between the groups (0.7 [CI 0.4–1.1]). When revision for aseptic loosening was studied as the endpoint, the survival for the study group was 99% (CI 98.6–99.4) and for the reference group 99% (CI 98.7–99.5), and the risk of revision was comparable between the study groups (HR 1.3 [CI 0.7–2.5]).

Interpretation — After an observation period of 7 years vitamin E-infused liners shows results equal to results obtained with crosslinked polyethylene liners.

Highly crosslinked polyethylene (HXLPE) was introduced in the late 1990s to decrease polyethylene wear and periprosthetic osteolysis and to increase the long-term survivorship of THA (Bragdon et al. 2013). HXLPE has shown lower wear rates in vitro (McKellop et al. 2000) and in vivo (Bragdon et al. 2013) compared with conventional non-crosslinked ultra-high-molecular-weight polyethylene (UHMWPE). However, as a downside, free radicals are released and oxidation is exacerbated, which may induce wear (Kurtz 2009). One potential solution to further decrease the number of free radicals in the liner material is to add vitamin E to HXLPE, which increases the resistance of polyethylene against these oxidative processes by stabilizing the material (Oral et al. 2006a, 2006b).

Since vitamin E-infused highly crosslinked polyethylene (VEPE) is a quite recent invention, there are only short- and mid-term data available on its efficacy and safety. Significantly lower femoral head penetration rates have been reported for VEPE liners by many authors in randomized controlled trials (RCTs) with radiostereometric analysis (RSA) compared with HXLPE liners (Nebergall et al. 2017, Scemama et al. 2017, Shareghi et al. 2017, Galea et al. 2018, Rochcongar et al. 2018, Sköldenberg et al. 2019). However, there is still a lack of real-world data from arthroplasty registers on the survival of VEPE liners.

We used the Finnish Arthroplasty Register (FAR) to assess implant survival of vitamin E-infused HXLPE liners (E1, E-poly, ZimmerBiomet, Warsaw, IN, USA) compared with moderately crosslinked polyethylene (ModXLPE) liners from the same manufacturer for revision for any reason. We further compared the study groups for those revisions performed for aseptic loosening, osteolysis, or polyethylene wear. We hypothesize that there is no statistically significant difference in outcome between the study groups.

## Patients and methods

Our study is based on data from the Finnish arthroplasty register (FAR), which has collected information on arthroplasties since 1980 (Paavolainen et al. 1991). The register acts nationwide, with data completeness exceeding 95% for primary THA and 81% for revision THA (FAR). The register is administered by the Finnish National Institute for Health and Welfare. Orthopedic units are obligated to provide information essential for maintenance of the register. Dates of death are obtained from the Population Information System maintained by the Population Register Center. The data content of the FAR was scrutinized and revised in May 2014. The updated data now includes more detailed information such as more precise reasons for revisions. Prior to the 2014 update, revisions for liner wear were recorded as performed “for other reason.”

### Study population

Between January 2000 and December 2017, 133,488 primary THAs were reported to the FAR. We included in the study group any THAs in which the vitamin E-infused HXLPE (E1, E-poly) liner option was used with 1 of 5 uncemented acetabular components from the same manufacturer (ZimmerBiomet): Biomex, Exceed, G7, Regenerex, and Vision Ringloc (Table 1). The reference group consisted of THAs used with ModXLPE liners from the same manufacturer (mostly ArCom) with the same cup designs. Exclusion criteria were head size other than 28 mm, 32 mm, or 36 mm; dual mobility acetabular device; metal or ceramic liner; or constrained liner. Only THAs with uncemented stems were included in the study. In 5,430 THAs the study inclusion criteria were fulfilled, and femoral head material information was available (2,723 E-poly or E1 liner THAs). The study group patients were operated on between January 1, 2008 and December 31, 2017, with a mean follow-up time of 5.0 years (0–9.7). The reference group patients were operated on between January 1, 2000 and December 31, 2017, with a mean follow-up time of 11.0 years (0–18.5). The number of patients with bilateral hip prostheses was 410, of whom 85 had both hips done simultaneously. Mortality during the study period was 10% in the VEPE group and 27% in the control group. Differences in mortality between the groups are explained by the difference in follow-up time (Table 2).

**Table 1.** Components used, number (%)

	VEPE group	Reference group
Cup designs		
Biomex **^a^**	0 (0)	56 (2)
Exceed **^a^**	506 (19)	312 (12)
G7 **^a^**	501 (18)	211 (8)
Regenerex **^a^**	741 (27)	6 (0.2)
Vision Ringloc **^a^**	975 (36)	2,122 (78)
Stem designs		
Bi-Metric **^a^**	1,143 (42)	2,459 (91)
CDH **^a^**	3 (0.1)	20 (0.7)
Echo **^a^**	1,357 (50)	174 (6)
Reach **^a^**	18 (1)	4 (0.2)
Taperloc **^a^**	202 (7)	50 (2)

**^a^** ZimmerBiomet, Warsaw, IN, USA.

**Table 2.** Demographic data of study population, number (%), unless stated otherwise

Data	VEPE group	Reference group
Mean age, years (SD)	67 (10)	64 (9)
BMI (SD)	29 (5)	28 (5)
Male sex	1,341 (49)	1,357 (50)
Diagnosis		
Primary osteoarthritis	2,328 (86)	2,274 (84)
Rheumatoid arthritis	59 (2)	83 (3)
Other **^a^**	336 (12)	350 (13)
Femoral head size		
28	4 (0.2)	2,229 (82)
32	321 (12)	284 (11)
36	2,398 (88)	194 (7)
Femoral head material		
Ceramic	822 (30)	220 (8)
Metal	1,901 (70)	2,487 (92)
Status at end of follow up **^b^**		
Not revised	2,571 (94)	2,348 (87)
Revised	152 (6)	359 (13)
Operation year		
2000–2008	6 (0.2)	2,376 (88)
2009–2017	2,717(99.8)	331 (12)

**^a^** Fractures, avascular necrosis, osteoarthritis due to hip dysplasia, tumors, congenital hip dislocation, Mb Legg–Calve–Perthes, femoral head epiphysiolysis.

**^b^** Excluding death.

### Surgery

In both groups, the most common cup design was Vision RingLoc (36% in the VEPE group, 78% in the control group). The most frequently used stems in the study population were Echo in the VEPE group (50% of all VEPE THAs) and Bi-Metric in the control group (91% of all THAs in the control group) (Table 1). A ceramic head was used in 30% of cases in the VEPE group compared with 8% in the reference group. In the VEPE group 36 mm femoral heads were used in 88% of cases, while 28 mm was the most commonly used head size in the control group (82%).

### Statistics

The primary outcome was revision for any reason and the secondary outcome was revision for loosening of the cup, osteolysis, liner wear, or liner breakage. Prior to the register update in 2014, revisions performed for osteolysis and wear were coded as performed for “other reason”; therefore, revisions performed for “other reason” prior to May 2014 are included in the analyses for secondary outcome. Patients were excluded for any other event than the outcome, or at the end of follow-up. Kaplan–Meier survival estimates with 95% confidence interval (CI) were calculated for both groups at 1, 3, 5, and 7 years for any reason for revision and for loosening of the cup, osteolysis, liner wear, or liner breakage. The survival curves were compared using the log-rank test. Revision was described as a change or removal of at least one component.

We adjusted the estimated revision risks in the Cox multiple regression model by sex, operated side, and femoral head material (ceramic, metal). Femoral head size (28, 32, 36 mm), age group (18–55, 56–65, 66–75, > 75 years), and preoperative diagnosis (primary osteoarthritis, rheumatoid arthritis, other) were stratified. None of these variables were considered to be along a causal pathway from exposure to outcome but were considered as confounders. The second analysis was performed for loosening of the cup, osteolysis, liner wear, or liner breakage as the endpoint. Side, femoral head material, sex, and diagnosis were adjusted for in the Cox model, and age group was stratified. Head size was excluded from this model because of large differences in head sizes between the groups.

If the proportional hazards assumption for a variable was not fulfilled in the Cox model, the model was stratified by it instead. Stratification in Cox models means that the hazard functions can be estimated for all level combinations of the stratified variables, and the hazard ratios for the other variables (those that meet the proportional hazard assumption) are then optimized for all these hazard functions. Without stratification we would assume that the hazards were the same for all levels of such variables. The results of the Cox regression analysis are presented with the hazard ratio (HR) and CI.

All analyses were performed using SAS software (Version 9.4; ASA Institute, Cary, NC. USA).

### Ethics, funding, and potential conflicts of interest

Ethical approval was from the Finnish National Institute for Health and Welfare (June 13, 2017, Dnor THL/926/5.05. 00/2017). This research received funding from the Finnish Government Research Grant. The authors declare no conflicts of interest.

## Results

### Revision for any reason

The 7-year survivorship with revision for any reason as endpoint was similar between the groups: 94.0% (CI 92.9–94.9) for the VEPE group and 93.0% (CI 91.9–93.9) for the reference group (Figure 2, Table 3, see Supplementary data). In the Cox regression analysis, the risk of revision in the VEPE group was lower, but the result was not statistically significant (HR 0.7 [CI 0.4–1.1]) (Table 4).

**Figure 1.**
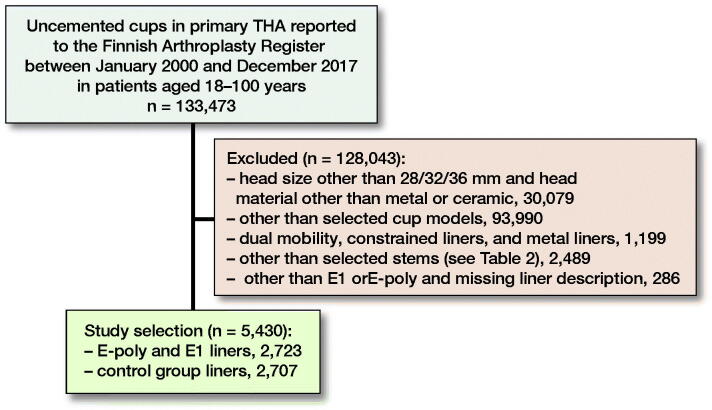
Flowchart of study selection.

**Figure 2.**
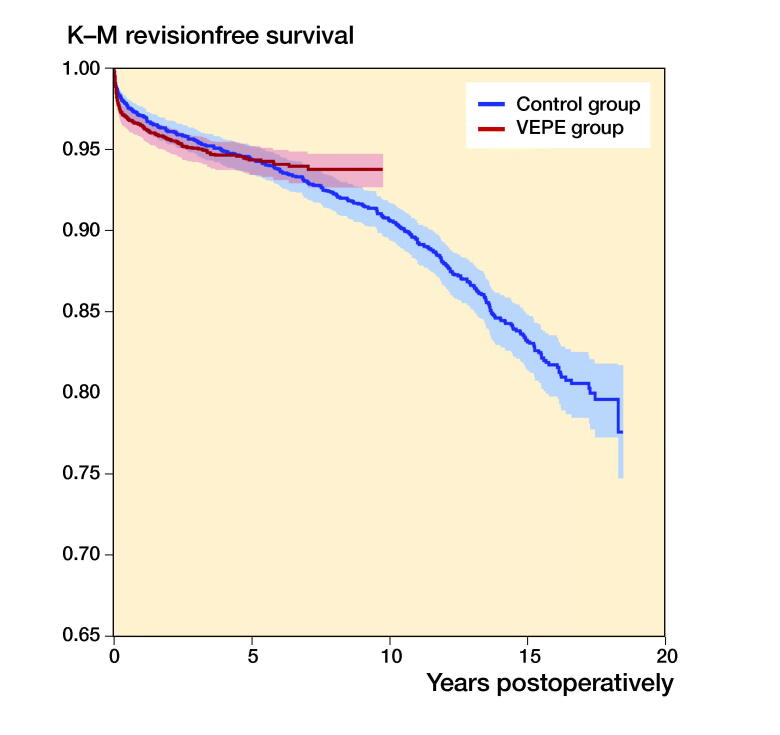
Kaplan–Meier survival for VEPE group and Reference group with revision for any reason as the endpoint. 95% CI levels presented in blue and red.

**Table 4.** Revision risk according to Cox regression model with all revisions as endpoint

Group	HR (95% CI)	p-value
VEPE group vs. Reference group	0.69 (0.44–1.1)	0.09
Adjusting variables		
Left vs. right side	0.98 (0.82–1.2)	0.8
Female vs. male	0.99 (0.83–1.2)	0.9
Ceramic vs. metal head	1.2 (0.90–1.5)	0.3

Adjusting variables stratified by head size, age group, and diagnosis. HR = hazard ratio. CI = confidence interval.

### Revision for aseptic loosening of the cup, osteolysis, liner wear, or liner breakage

**Figure 3.**
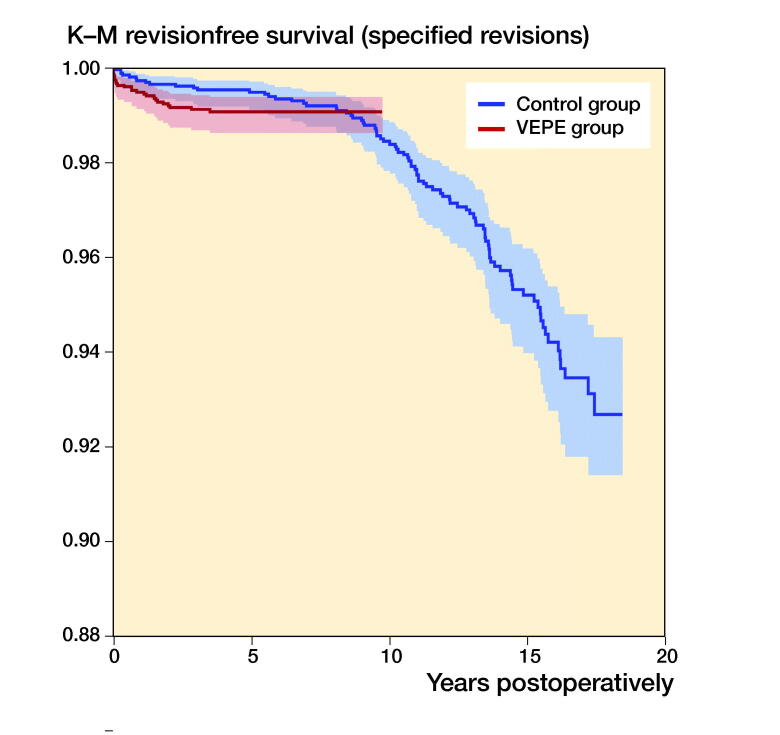
Kaplan–Meier survival for VEPE group and Reference group with revision for osteolysis, liner wear, liner breakage, loosening of the cup, and other reason as an endpoint. 95% CI levels presented in blue and red.

The 7-year survivorship with revision for aseptic loosening of the cup, osteolysis, liner wear, or liner breakage as endpoint was equal between the groups: (VEPE group 99.1% [CI 98.6–99.4]); reference group 99.2% (CI 98.7–99.5) (Figure 3, Table 5, see Supplementary data). The risk of revision in the VEPE group was the same as in the reference group (HR 1.3 [CI 0.7–2.5]) (Table 6).

**Table 6.** Revision risk according to Cox regression model with revision for osteolysis, liner wear, liner breakage, loosening of the cup (and other reason before May 15, 2014) as endpoint

Group	HR (95% CI)	p-value
VEPE group vs. Reference group	1.3 (0.71–2.5)	0.4
Adjusting variables		
Female vs. male	1.0 (0.71–1.5)	0.9
Other diagnoses vs. RA	0.96 (0.35–2.6)	0.9
Primary OA vs. RA	0.85 (0.34–2.1)	0.7
Ceramic vs. metal head	1.1 (0.70–1.8)	0.6

Adjusting variables are stratified by age group and side.

HR = hazard ratio. CI = confidence interval, OA = osteoarthritis,

RA = rheumatoid arthritis.

**Table 7.** Indication for revision prior to data content revision (May 15, 2014) of Finnish Register, number (%)

Main reason for revision **^a^**	VEPE group	Reference group
Aseptic loosening		
Cup and stem	0 (0)	2 (1)
Cup	9 (10)	10 (4)
Stem	2 (2)	9 (4)
Infection	10 (11)	17 (7)
Dislocation	27 (30)	103 (46)
Component malposition	7 (8)	22 (10)
Fracture	24 (26)	23 (10)
Component breakage	0	2 (1)
Other	12 (13)	41 (18)

**^a^** No data available concerning indication for revision from 36 revisions.

### Reasons for revision

The most frequent reason for revision before the register update (May 2014) were dislocation (27%), periprosthetic fracture (24%), and infection (11%) in the VEPE group, and dislocation (46%), other reason (18%), and component malposition (10%), as well as periprosthetic fracture (10%) in the reference group (Table 7). After the register update the most frequent reason for revision was dislocation (33%), followed by infection (21%), and periprosthetic femoral fracture (14%) in the VEPE group, and dislocation (23%), liner wear (22%), and periprosthetic femur fracture (17%) in the reference group (Table 8). Liner breakage was observed in 2 patients in the VEPE group and 3 patients in the reference group in the scrutinized register data (5% VEPE group, 3% reference group).

**Table 8.** Indication for revision after new indications for revision were added at the data content revision: data starting from May 15, 2014, number (%)

Main reason for revision **^a^**	VEPE group	Reference group
Aseptic loosening		
Cup	1 (2)	3 (3)
Stem	3 (7)	2 (2)
Osteolysis		
Cup	0	11 (12)
Stem	0	3 (3)
Liner wear	0	20 (22)
Component breakage		
Cup	0	1 (1)
Liner	2 (5)	3 (3)
Modular neck	0	0
Infection	9 (21)	5 (6)
Dislocation	14 (33)	21 (23)
Component malposition		
Cup	2 (5)	4 (4)
Periprosthetic fracture		
Acetabulum	0	1 (1)
Femur	6 (14)	15 (17)
Unexplained pain	1 (2)	0
Leg length discrepancy repair	2 (5)	0
Other	3 (6)	1 (1)

**^a^** No data available concerning indication for revision from 27 revisions.

## Discussion

We found that VEPE liners perform comparably to ModXLPE liners at mid-term follow-up. The risk of revision in the VEPE group was lower when revision for any reason was the end point but the result was not statistically significant (HR 0.7 [CI 0.4–1.1]). This is one of the largest studies of VEPE liners with a mean follow-up of 5 years. Our findings support the assumption that VEPE liners are durable and safe; however, further studies with longer follow-up are needed to assess the long-term survival and possible benefits of this material (Sillesen et al. 2016, Nebergall et al. 2017, Galea et al. 2019).

Charnley first introduced ultrahigh molecular weight polyethylene (UHMWPE) in 1960 as the bearing material for the recently developed THA. The sterilization of conventional polyethylene (PE) was performed by gamma irradiation in air. The benefit of this process is molecular crosslinking, which provides improved wear resistance. On the downside, this process produces free radicals that decrease resistance and cause degradation and thus increase polyethylene wear (McKellop et al. 2000). PE debris may induce periprosthetic osteolysis through the release of cytokines and proteolytic enzymes and thus wear of bearing surfaces is thought to be the main limiting factor of long-term survival of THA (Merola and Affatato 2019).

HXLPE was introduced in the late 1990s to decrease polyethylene wear and osteolysis (Bragdon et al. 2013). Today, it is considered the gold standard for acetabular liners in THA (Oral et al. 2007). The number of free radicals formed in the crosslinking procedure can be reduced by heating the material above its melt temperature or annealing below its melt temperature after crosslinking (Baker et al. 2003). However, the processes do not eliminate all free radicals (Currier et al. 2007, Kurtz 2009). VEPE liners were developed to further improve the oxidative stability of radiated XLPE. The added vitamin E increases the resistance of polyethylene against oxidative processes by stabilizing the material (Oral et al. 2006a, 2006b). VEPE has shown excellent wear characteristics and resistance to oxidative stress in laboratory conditions (Oral et al. 2006a). There are generally 2 methods of adding vitamin E to crosslinked UHMWPE: blending the vitamin E with the UHMWPE before irradiation and crosslinking or infusing it after crosslinking (Rowell et al. 2011). A higher vitamin E concentration can be achieved by infusing it into the HXLPE (Rowell et al. 2011), but the clinical effect is unclear (Kurtz et al. 2009).

Liner wear is often assessed by measuring the penetration of the femoral head into the liner with RSA. However, the real penetration rate comprises not only the true loss of PE but also creep deformation of the liner. Several RCTs have been performed comparing femoral head penetration rates of VEPE and ModXLPE liners using RSA. Some authors have reported lower penetration rates in VEPE patients at short- to medium-term follow-up, although wear rates have been low in both groups (Salemyr et al. 2015, Scemama et al. 2017, Shareghi et al. 2017, Galea et al. 2018, Rochcongar et al. 2018). Almost as many authors have reported equal penetration rates at medium-term follow-up (Nebergall et al. 2017, Galea et al. 2019, Busch et al. 2020). Lindalen et al. (2019) compared 32 mm versus 36 mm ceramic femoral heads with VEPE liners and did not find any differences in wear rates in RSA measurements at 6-year follow-up. The wear rates have been low in both groups and well below the reported osteolytic threshold of 0.1 mm/year (Dumbleton et al. 2002); therefore, the measured statistically lower penetration rates might not be clinically relevant, and longer follow-up is needed.

The Australian Orthopaedic Association National Joint Replacement Registry (AOANJRR) has reported a similar revision risk for THAs with antioxidant inserts compared with ModXLPE inserts (AOANJRR 2019). The 8-year Kaplan–Meier estimates (cumulative percent revision) of 6,046 conventional THAs using the Ringloc cup with XLPE or VEPE liners were 2.5 for both groups (2.5 [CI 2.0–3.2]) and 2.5 [CI 1.9–3.1], respectively). The 4-year Kaplan–Meier estimate for 2,729 THAs using the G7 cup with VEPE liner was inferior to that for the XLPE liner (1.8 [CI 1.3–2.5]) and 2.9 [CI 1.3–6.3]), respectively), although the number of XLPE liners was limited. Recently published work based on the National Joint Registry (NJR) found that VEPE liners, HXLPE liners (radiation ≥ 5 mrad), and liners heated above the melting point were associated with best survival in a cohort of 292,920 primary THAs. For VEPE liners, the 8-year cumulative incidence function of revision due to aseptic loosening was 0.3 and due to reasons other than aseptic loosening 1.7 (values estimated from the figure). However, the follow-up time of 11,926 VEPE liners was relatively short (3.3 years) (Davis et al. 2020). A multinational collaboration study of 977 patients reported equal performance between the VEPE liner and ModXLPE liner at 3-year follow-up, and no early in-vivo adverse effects were observed (Sillesen et al. 2016). Our findings support these earlier findings. Our study design was to compare the same cup brands from the same manufacturer with either a VEPE or ModXLPE liner. We think this is an optimal study setting to compare differences between these liner materials as cup designs do not bias the results. The reference group consist of ModXLPE liners whereas VEPE liners are made of HXLPE. The amount of cross-linking and thus wear resistance increases with increasing radiation dose (McKellop et al. 1999), but higher doses are also associated with a decrease in tensile and fracture toughness (Gomoll et al. 2002). All in all, a recent large register study of 292,920 primary THAs did not find any difference in the survival of moderately and highly irradiated liners at maximum follow-up of 14 years (Davis et al. 2020).

Prior to the Finnish register revision of 2014, liner wear was not recorded separately but as “other reason,” which may cause minor bias. The proportion of revisions performed for loosening and wear in our study is in line with other registers (AOANJRR 2019). There were 2 revisions performed due to liner breakage in the VEPE group versus 3 revisions in the reference group after 2014, accounting for 5% of revisions in the VEPE group and 3% in the reference group. Reports of VEPE liner breakage in the literature are rare (Bates and Mauerhan 2015, Brazier and Mesko 2018), and current data support the previous findings. Concerns over safety issues have not been raised in several previous studies, and our results are in agreement with this (Jarrett et al. 2010, Gigante et al. 2015, Sillesen et al. 2015, Davis et al. 2020).

The primary strength of this nationwide study is the large population-based setup with a mean 5-year follow-up time for the VEPE group. A limitation of the study is that we were not able to assess radiographs to evaluate wear. Further, we were not able to assess patient comorbidities (e.g., cardiovascular diseases, psychiatric disorders, and cancer) which could affect revision rates. Revision operation was also the only outcome we were able to assess, as FAR data contents do not include patient-reported outcome measures. The study groups were operated on in somewhat different time eras. However, at the same time, this is also a strength of our study, as we wanted in particular to assess two generations of liner materials from the same manufacturer using the same acetabular components. Femoral head size increased so substantially during the study period that we were not able to use it as a variable in the Cox model with osteolysis and wear as the endpoint (wide confidence intervals). The portion of ceramic heads between the groups was somewhat different (30% VEPE group versus 8% reference group), but a recent study found similar wear rates between metal and ceramic heads (Gaudiani et al. 2018). Despite these weaknesses of our study, we do not feel that our message is undermined and VEPE liners are a safe option with good medium-term results.

In conclusion, after an observation period of 7 years, vitamin E-infused liners show results equal to results obtained with crosslinked polyethylene liners and results are in line with previous findings. Longer follow-up is needed to assess the potential advantages, if any, of VEPE liners in the long term.

## Supplementary data

Tables 3 and 5 are available as supplementary data in the online version of this article, http://dx.doi.org/10.1080/17453674. 2021.1879513
